# Scores of Brunei Lower Secondary School Students on Emotional Intelligence Variables: Exploring the Differences

**DOI:** 10.5539/gjhs.v8n4p22

**Published:** 2015-07-30

**Authors:** Norfaezah Masri

**Affiliations:** 1 Sultan Hassanal Bolkiah Institute of Education, Universiti Brunei Darussalam, Jalan Tungku Link, Gadong BE 1410, Bandar Seri Begawan, Brunei Darussalam

**Keywords:** emotional intelligence, adolescent, secondary school, coping

## Abstract

The survey compared the emotional intelligence of 254 (128 females) randomly selected Year 11 Brunei Cambridge General Certificate of Education (BCGCE) Ordinary Level students using the six subscales of the BarOn Emotional intelligence scale – youth version. Females scored significantly higher on the intrapersonal variable than males. However, males sored much higher on the positive impression subscale. In addition, students aged 16 scored significantly higher on the interpersonal scale than all others. However, the 15-year olds scored highest on the adaptability and positive impression scales than their peers. Furthermore, participants who reported that they were not so much satisfied with their personal life scored significantly higher on the interpersonal scale than their counterparts. Moreover, participants who consult friends when faced with problems scored significantly higher on the interpersonal variable while those who search the internet for solutions to problems scored higher than others on the adaptability scale. No significant differences were obtained on any subscale when participants were compared on the basis of their parents’ marital status as well as the type of guardian they stayed / lived with. Implications of the findings are discussed and mixed-methods research was recommended.

## 1. Introduction

Emotional Intelligence (EI) is a distinctive social intelligence that involves the aptitude to monitor one's own and other's feelings and emotions to distinguish among them and to use the information to guide one's own thinking and actions (Bar-On & Parker, 2000; Salovey, Brackett, & Mayer, 2004). At one point in time, Intelligence Quotient (IQ) was viewed as the primary determinant of success. People with high IQs were assumed to be destined for a life of accomplishment and achievement and previous researchers have debated whether IQ was the product of genes or the environment or both. People with high IQs typically do well in school. However, today experts recognize it is not the only determinant of life success. Instead, it is part of a complex array of influences that include emotional intelligence among other things (Cherry, 2014).

Salovey (2003) defined EI as “the ability to perceive and express emotion, assimilate emotion in thought, understand and reason with emotion, and regulate emotion in the self and others.” According to Goleman (1995) EI consists of five components: knowing your emotions (self-awareness), managing them, motivating ourselves, recognizing emotions in others (empathy), and handling relationships. Segal and Smith (2014) version of EI is “the ability to identify, use, understand, and manage emotions in positive ways to relieve stress, communicate effectively, empathize with others, overcome challenges, and defuse conflict.” EI is the area of cognitive ability involving traits and social skills that facilitate interpersonal behavior. Intelligence can be broadly defined as the capacity for goal-oriented adaptive behavior; emotional intelligence focuses on the aspects of intelligence that govern self-knowledge and social adaptation (Rouse, 2010). Ultimately, being emotionally and socially intelligent means to effectively manage personal, social and environmental change by realistically and flexibly coping with the immediate situation, solving problems and making decisions. To do this, we need to manage emotions so that they work for us and not against us, and we need to be sufficiently optimistic, positive and self-motivated (Bar-On, R., 2006).

### 1.1 Research on Psychological Wellbeing of Brunei Secondary School and Tertiary Students

Studies that investigate the psychological and mental health states of Brunei secondary school students were still rare due to a variety of problens. Finding suitable research instruments is one of the main problems facing educational researchers in Brunei. Most of the good instruments are written in advanced English and many tend to be too long (Mundia & Bakar, 2010; Mundia, 2011). However, one related recent study that dealt with Brunei student teachers of different personality-orientation found that the trainee teachers had psychological problems of an emotional nature such as depression, anxiety and stress (Mundia, 2010a, 2010b). There is also evidence from research that depression, anxiety and stress were experienced by Brunei secondary school students due to a variety of problems they face in their everyday life (see Hamid et al., 2013; and Matzin et al., 2013). Quantitative subjects (such as mathematics, statistics, econometrics, or psychometrics) and English language are some of the courses or disciplines that students in the Brunei education system find challenging and distressful although they enjoy studying these subjects (Shahrill et al., 2013; Keaney & Mundia, 2014). If these emotionally-loaded personal problems were prevalent in small groups of Brunei trainee teachers and secondary school students, they may by extrapolation, also be numerous and common in the general Brunei society or population. Often, students do not know how to resolve their academic and personal problems satisfactorily and rarely sought help from mental health professionals such as school counselors and school psychologists (Mundia, 2010c, 2013; Shahrill & Mundia, 2014). Lack of emotional skills to resolve problems peacefully sometimes leads to physical fighting between the student and teacher (Mundia, 2006). Students who either drop out of school early or leave school early also need assistance to determine suitable careers for them so as to prevent them from becoming deliquents (Mundia, 1998). Some of the problems that occur at the lowest level of education such as the preschool, need to be resolved jointly by teachers and parents (Mundia, 2007). These findings suggest that there might be some sort of relationship between personality and other attributes such as EI. Under the ongoing SPN21 educational reforms in Brunei, teachers are required to play a central role in assessing learning not only quantitatively but also qualitatively by examining students’ personal traits that might impact learning either positively or negatively (Mundia, 2010d). In view of this, Brunei teacher education was also reformed to ensure that trainee teachers received adequate psychometric skills required in assessing students both qualitatively and quantitatively (Mundia, 2012). Furthermore, efforts are also currently being made to prepare teachers who have high self-efficacy in special education (Bradshaw & Mundia, 2005; Bradshaw & Mundia, 2006; Haq & Mundia, 2012; Tait & Mundia, 2012a; Tait & Mundia, 2013). Research has also revealed that Brunei trainee teachers of both genders have good interpersonal trust and interaction skills that may be helpful in assisting emotionally disturbed students with high support needs (Mahalle et al., 2013). Good social skills are essential in all ideal teachers (Omar et al., 2014). Since children are brought up in under two environments, home and school, research suggests that parents of children with challenging emotional behaviors should also receive training in handling effectively the emotions of children (Tait & Mundia, 2012b; Tait et al., 2014a; Tait et al., 2014b).

### 1.2 Objectives of the Study

Research has shown that EI is a better predictor of a student's academic performance than the more traditional measures of cognitive intelligence (Hettich, 2000). EI is one such factor which is instrumental in situations that call upon students to adjust successfully from one environment to another (Hettich, 2000). The purpose of the present study was to determine the extent or degree to which Brunei Year 11 students’ EI scores differ by demographical factors such as gender, age, student's satisfaction with her/his life, marital status of parents, source of help when faced with distress, and type of guidian they stayed/lived with.

## 2. Method

The design, sample, instruments, data analyses, and procedures used used are briefly described in Sections 2.1-2.5.

### 2.1 Design

The study used the field survey method to collect data to address the research objectives. The research approach allowed the investigator to involve many Year 11 students in the study.

### 2.2 Sample

Participant students were selected randomly. The researcher invigilated the students so that the questionnaires could be collected on the spot. The researcher also made sure that the students checked and answered all the items prior to collection to ensure getting a good number of usable returns. The participants’ bio-data (gender and age) are presented in Table 1.

**Table 1:** Demographic information (N=254)

Variable	Group	Frequency	Percentage
Gender	Males	126	49.6
	Females	128	50.4

Age	Group	Mean	SD

	All	16.602	0.740
	Males	16.698	0.707
	Females	16.507	0.763

### 2.3 Instruments

The researcher used the BarOn Emotional Quotient Inventory: Youth Version (BarOn EQ-i: YV) to collect the research data. The BarOn EQ-i: YV (BarOn & Parker, 2000) consists of 60 items that are distributed across six scales: Intrapersonal (6 items); Interpersonal (12 items); Stress Management (12 items); Adaptability (10 items); General Mood (14 items); and, Positive Impression (6 items). Each item is rated on a 4-point Likert-type scale ranging from 1 = Very seldom true of me, 2 = Seldom true of me, 3 = Often true of me, and, 4 = Very often true of me.

The interpretations and meanings attached to criterion scale scores are as follows:

- Intrapersonal: less than a score of 20 suggests that these individuals may not understand their emotions and are not able to express and communicate their feelings and needs
- Interpersonal: less than a score of 35 suggests that these individuals are likely to have satisfying interpersonal relationships. They are good listeners and are able to understand and appreciate the feelings of others.
- Stress Management: less than a score of 30 suggests that these individuals are generally calm and work well under pressure. They are rarely impulsive and can usually respond to a stressful event without an emotional outburst.
- Adaptability: less than a score of 30 suggests that these individuals are flexible, realistic, and effective in managing change. They are good at finding positive ways of dealing with everyday problems.
- General Mood: less than a score of 35 suggests that these individuals are optimistic. They also have a positive outlook and are typically pleasant to be with.
- Positive Impression: less than a score of 20 suggests that these individuals may be attempting to create an overly positive self-impression.

The descriptive statistics and reliability of the instruments are presented in Table 2.

**Table 2:** Descriptive statistics and reliability of the instrument (N = 254)

Scales		Items	Means	SE Mean	SD	Alpha
Intrapersonal		6	14.283	0.218	3.484	0.698
Interpersonal		12	33.299	0.478	7.623	0.865
Stress Management		12	30.677	0.379	6.044	0.742
Adaptability		10	26.547	0.295	4.703	0.733
General Mood		14	35.889	0.491	7.831	0.867
Positive Impression		6	14.984	0.175	2.791	0.640

The correlations in Table 3 may be interpreted in many ways. The low and non-significant correlations suggest that the paired scales assessed different constructs and did not replicate each other. For these scales, the correlations provide good quantitative evidence for the discriminant validity of the scales. The low but significant correlations imply that the scales (to a small extent) might be overlapping and measuring the same construct but the amount of duplication or common variance is little and negligible. The paired scales can thus be said to have satisfactory discriminant validity and low convergence validity. The high and significant positive correlations (0.500-0.700 and abobe) suggest that the scales concerned had good convergence/concurrent validity.

**Table 3:** Convergence and discriminant validity of the instruments (N = 254)

Scale†	A	B	C	D	E
A	1				
B	-0.165**	1			
C	-0.096	0.095	1		
D	-0.009	0.583**	-0.042	1	
E	-0.071	0.730**	0.238**	0.552**	1
G	0.269**	0.185**	-0.006	0.362**	0.365**

** p < .01 (two-tailed)

† [A – Intrapersonal Scale, B – Interpersonal Scale, C – Stress Management Scale.

D – Adaptability Scale, E – General Mood Scale, G – Positive Impression Scale].

### 2.4 Data Analysis

The quantitative data were analyzed by both descriptive statistics (frequencies, percentages, mean and standard deviation) and inferential statistics (t-test for independent samples incorporating ANCOVA F, Pearson's correlation, and One-Way ANOVA including Eta squared values). The rationale and justification for using these techniques is two-fold. First, the procedures were deemed to be appropriate for addressing the research objectives. Second, the data were obtained from a random sample and there was no evidence of violation of the statistical assumptions.

### 2.5 Procedures

Prior to collecting the data, a permission to conduct the study in schools was obtained from the Ministry of Education in the Government of Brunei. The participants were told about the purpose and the objectives of the study. No dishonesty was involved in the study. The participants were told verbally about the ethical conditions or requirements for being involved in the study. The discussion on this topic centered on issues of voluntary participation, privacy, anonymity, confidentiality, physical and psychological harm, debriefing, and informed consent. Students were given ample time to reflect on and withdraw from the study if they felt uncomfortable about participating. With regard to English language problems, the meanings of difficult English words, sentences and phrases on the instruments were verbally explained to the participants. The researcher therefore considered it not necessary to translate the instruments into Bahasa Melayu (Brunei's main and official language). Although most participants required only 30 minutes to complete the instruments, those with reading problems were allowed longer time.

## 3. Results

The findings are presented below under separate subheadings.

### 3.1 Differences by Gender

Females scored significantly higher on the intrapersonal variable than males (Table 4). However, males sored much higher on the positive impression subscale (Table 4).

**Table 4:** Differences by gender (N=254)

Factors	Male (n=126)		Female (n=128)		ANCOVA	T	P	ES
(Scales)	Mean	SD	Mean	SD	F	(*df*=252)	(2-tailed)	
A	14.063	2.927	14.500	3.956	12.726***	-0.998	0.319	0.063
B	33.269	7.419	33.328	7.848	0.771 ns	-0.061	0.952	0.004
C	31.357	6.393	30.007	5.625	1.836 ns	1.787	0.075	0.112
D	26.801	4.710	26.296	4.701	0.000 ns	0.855	0.394	0.054
E	37.190	7.736	34.609	7.742	0.567 ns	2.658	0.008	0.165
G	15.230	2.614	14.742	2.946	3.853*	1.395	0.164	0.088

[A – Intrapersonal Scale, B – Interpersonal Scale, C – Stress Management Scale, D – Adaptability Scale, E – General Mood Scale, G – Positive Impression Scale].

### 3.2 Differencs by Age

According to Tukey HSD, students aged 16 scored significantly higher on the interpersonal scale than all the others (Table 5). The 15-year olds scored highest on the adaptability and positive impression scales than others (Table 5).

**Table 5:** Differences by age (N=254)

Factors	Age 15	Age 16	Age 17	Age 18	F	P	Eta
	(n=10)	(n=110)	(n=105)	(n=29)	(*df*=3)	(2-tailed)	

(Scales)	Mean (SD)	Mean (SD)	Mean (SD)	Mean (SD)			
A	15.100	14.027	14.581	13.896	0.753	0.521	0.095
	(4.067)	(3.905)	(3.115)	(2.820)			
B	32.000	34.609	32.933	30.103	3.030	0.030*	0.187
	(8.819)	(7.625)	(7.395)	(7.222)			
C	29.800	31.109	30.438	30.206	0.368	0.776	0.066
	(5.633)	(6.481)	(5.982)	(4.693)			
D	29.200	26.845	26.552	24.482	3.148	0.026*	0.191
	(3.119)	(5.142)	(4.033)	(5.110)			
E	38.100	36.463	36.000	32.551	2.259	0.082	0.162
	(7.475)	(7.833)	(7.557)	(8.398)			
G	16.500	15.236	14.990	13.482	4.234	0.006**	0.220
	(2.273)	(2.908)	(2.431)	(3.236)			

* p < .05 (2-tailed)

** p < .01 (2-tailed).

[A – Intrapersonal Scale, B – Interpersonal Scale, C – Stress Management Scale, D – Adaptability Scale, E – General Mood Scale, G – Positive Impression Scale].

### 3.3 Differences by Satisfaction With Personal Life

Participants who reported that they were not so much satisfied with personal life scored significantly higher on the interpersonal scale than the others (see Table 6). There were no other significant differences.

**Table 6:** Differences by satisfaction with personal life (N=254)

Factors	Very much	Not so much	Little	F	P	Eta
	(n=22)	(n=122)	(n=110)	(*df*=2)	(2-tailed)		

(Scales)	Mean (SD)	Mean (SD)	Mean (SD)						
A	14.590	14.204	14.309	0.119	0.888	0.031
	(3.275)	(3.622)	(3.393)			
B	29.590	33.139	34.218	3.497	0.032*	0.165
	(7.142)	(7.646)	(7.516)			
C	30.181	30.262	31.236	0.831	0.437	0.081
	(6.814)	(5.516)	(6.443)			
D	24.727	26.336	27.145	2.695	0.069	0.145
	(3.088)	(4.854)	(4.719)			
E	32.000	35.016	37.636	6.477	0.002**	0.222
	(5.789)	(7.228)	(8.42)			
G	13.409	14.672	15.645	7.741	0.001**	0.241
	(2.938)	(2.54)	(2.865)			

* p <0.05 (2-tailed)

** p < .01 (2-tailed).

[A – Intrapersonal Scale, B – Interpersonal Scale, C – Stress Management Scale, D – Adaptability Scale, E – General Mood Scale, G – Positive Impression Scale].

### 3.4 Differences by Parents’ Marital Status

No significant differences were obtained on this factor as indicated in Table 7.

**Table 7:** Differences by parents’ marital status (N=254)

Factors	Single	Married	Divorced	Widow	F	P	Eta
	(n=5)	(n=219)	(n=26)	(n=4)	(*df=*3)	(2-tailed)		

(Scales)	Mean (SD)	Mean (SD)	Mean (SD)	Mean (SD)						
A	15.000	14.219	14.538	15.250	0.242	0.867	0.054
	(2.738)	(3.55)	(2.983)	(4.573)			
B	31.800	33.301	32.961	37.250	0.437	0.727	0.072
	(3.701)	(7.642)	(8.204)	(7.228)			
C	29.800	30.648	30.807	32.500	0.160	0.923	0.044
	(3.271)	(5.991)	(7.31)	(2.645)			
D	25.800	26.557	26.230	29.000	0.441	0.724	0.073
	(2.588)	(4.687)	(5.171)	(5.228)			
E	39	35.666	36.230	42.000	1.152	0.329	0.117
	(1.870)	(7.889)	(8.086)	(5.416)			
G	16.200	14.844	15.884	15.250	1.419	0.238	0.129

[A – Intrapersonal Scale, B – Interpersonal Scale, C – Stress Management Scale, D – Adaptability Scale, E – General Mood Scale, G – Positive Impression Scale].

### 3.5 Differences by Sources of Help When Distressed

Participants who consult friends when faced with problems scored significantly higher on the interpersonal variable than the others (Table 8). Those who search the internet for solutions to problems scored higher than others on the adaptability scale (Table 8).

**Table 8:** Differences by source of help (N=254)

Factors	Family	Prayers	Yourself	Friends	Internet	Other	F	P	Eta
	(n=80)	(n=59)	(n=54)	(n=42)	(n=11)	(n=8)	(*df*=5)	(2-tailed)		

(Scales)	Mean (SD)	Mean (SD)	Mean (SD)	Mean (SD)	Mean (SD)	Mean (SD)						
A	14.537	13.762	14.407	14.881	14.000	12.000	1.319	0.256	0.161
	(3.325)	(3.554)	(3.647)	(3.415)	(3.162)	(3.817)			
B	31.525	35.186	32.000	35.642	32.727	34.375	2.841	0.016*	0.233
	(7.577)	(6.986)	(8.018)	(7.505)	(6.466)	(7.595)			
C	30.050	32.101	30.777	29.119	33.272	30.375	1.828	0.108	0.189
	(4.854)	(5.668)	(6.433)	(6.783)	(7.295)	(9.085)			
D	25.587	26.762	26.314	27.381	30.454	26.547	2.580	0.027*	0.222
	(4.154)	(4.095)	(5.344)	(4.411)	(4.885)	(4.703)			
E	34.275	38.101	35.074	36.238	38.454	35.875	2.032	0.075	0.198
	(7.655)	(7.063)	(7.686)	(8.400)	(8.664)	(9.311)			
G	14.487	15.016	15.074	15.381	15.818	15.875	1.049	0.389	0.144
	(2.774)	(2.763)	(2.662)	(3.059)	(2.088)	(3.313)			

* P<0.05 (2-tailed).

[A – Intrapersonal Scale, B – Interpersonal Scale, C – Stress Management Scale, D – Adaptability Scale, E – General Mood Scale, G – Positive Impression Scale].

### 3.6 Differences by Type of Guardian They Stay/Live With

No significant differences were found on this factor as shown in Table 9.

**Table 9:** Differences by whom they stay/live with (N=254)

Factors	Parents	Mother	Other	F	P	Eta
	(n=219)	(n=22)	(n=13)	(*df*=2)	(2-tailed)	

(Scales)	Mean (SD)	Mean (SD)	Mean (SD)			
A	14.205	14.318	15.538	0.898	0.408	0.084
	(3.543)	(2.868)	(3.406)			
B	33.278	34.409	31.769	0.494	0.611	0.063
	(7.545)	(8.033)	(8.555)			
C	30.684	31.409	29.384	0.457	0.634	0.06
	(5.973)	(6.456)	(6.801)			
D	26.529	26.818	26.384	0.450	0.956	0.019
	(4.664)	(5.653)	(3.884)			
E	35.748	37.545	35.461	0.545	0.581	0.066
	(7.860)	(8.140)	(6.995)			
G	14.872	15.818	15.461	1.352	0.261	0.103
	(2.802)	(3.289)	(0.967)			

[A – Intrapersonal Scale, B – Interpersonal Scale, C – Stress Management Scale, D – Adaptability Scale, E – General Mood Scale, G – Positive Impression Scale].

## 4. Discussion

The present study found that there were not many significant differences between Brunei male and female secondary school students in terms of emotional intelligence. Several theorists have previously pointed out the importance of the ability to differentiate among these feelings, and to normalize one's feelings as facades of emotional processing that are important in order to adaptively use the information conveyed by one's emotions and enhance psychological well-being (Heck & Oudsten, 2008; Salovey et al., 2004). According to the literature, adolescents with high expectations regarding their ability to understand and manage their moods experience and savor more positive emotions and tend to be more satisfied with their life (Salovey et al., 2009). Similarly, feelings of self-worth and self-acceptance play an important role in the process of psychological adaptation and emotional well-being in adolescents (Leary, 1999). The findings of the present study may be beneficial in developing more empirically-authenticated positive psychology intervention programs to facilitate well-being in young adolescents. Emotionally intelligent adolescents might be taught how to employ specific strategies for repairing negative moods and increasing positive ones to help them to increase their feelings of self-worth. This in turn might assist them in increasing feelings of satisfaction with their life during adolescence. Understanding adolescents in all their diversity is fundamental to improving their lives. Adolescents experience intense physical, psychological, emotional and economic changes as they make the transition from childhood to adulthood. Risk-taking is part of adolescence, and it is the obligation of society both to prevent risk and to moderate any audacious consequences such risk-taking was bound to have.

Adolescence is a developmental period during which children grow into their rightful place as full citizens and agents of change in their own lives and the lives of their societies. As they physically and psychologically mature, they form their values, core beliefs, sense of identity and understanding their place in the world. Adolescence was also a time when children's and young people's relationships with the people and communities that surround them can change dramatically. They leave behind childhood and take on new roles such as earners, holders of adult rights, and duty bearers in their communities and societies. Thus adolescents must be valued as an asset to society. It is time to invest in them to ensure that they have the opportunity to fully realize their potential and that of their communities - a future in which adolescents are healthy, educated, protected, and empowered (UNICEF, 2012).

## 5. Conclusion

A few significant diffrences were found among the participants on some demographical factors and EI variables as indicated in results section above (Tables 4-9). The identified EI variables and demographical factors could be included into intervention workshops and counselling sessions for needy students both individuals and groups. The relationship between students and teachers and the interactions between students and the school environment all have direct effects on learning and behavior.

## 6. Limitations

The study had three main limitations. First, due to time constraints, only 254 randomly selected students were used as participants. Thus the relatively small sample did not represent all the secondary students’ emotional intelligence in Brunei. Second, as a survey, the results of the present study could not establish cause-effect relationships among the variables investigated. Third, a qualitative interview component was missing but necessary to supplement findings from the quantitative survey. The findings imply that the present study was worth replicating to confirm the results.
